# Injury Characteristics and Prognostic Outcomes of Ankle Fracture–Dislocation: The Importance of Posteromedial Extension of Posterior Malleolar Fractures

**DOI:** 10.3390/jcm15166231

**Published:** 2026-08-12

**Authors:** Ji Seong Park, Byung Ki Cho, Tae Kyun Kim, Chan Kang, Gi Soo Lee, Jae Hwang Song

**Affiliations:** 1 Department of Orthopaedic Surgery, Konyang University Hospital, Daejeon 35365, Republic of Korea; jiseong9612@gmail.com (J.S.P.); ktk1113@hanmail.net (T.K.K.); 2 Department of Orthopaedic Surgery, College of Medicine, Konyang University, Daejeon 35365, Republic of Korea; 3 Department of Orthopaedic Surgery, Chungbuk National University Hospital, Chungbuk National University College of Medicine, Cheongju 28644, Republic of Korea; titanick25@naver.com; 4 Department of Orthopaedic Surgery, College of Medicine, Chungnam National University, Daejeon 35015, Republic of Korea; faschan@hanmail.net (C.K.); gs1899@gmail.com (G.S.L.)

**Keywords:** ankle, fracture, dislocation, posterior malleolar, syndesmosis, risk factor

## Abstract

**Background/Objectives:** Ankle fracture–dislocation is associated with prolonged recovery, posttraumatic osteoarthritis, and poor functional outcomes. However, the structural factors responsible for ankle fracture–dislocation remain incompletely understood. This study aimed to identify the injury characteristics associated with ankle fracture–dislocation and compare radiographic outcomes between patients with and without dislocation. **Methods:** A retrospective review was performed on 130 patients who underwent operative treatment for ankle fractures. Patients were divided into dislocation (*n* = 26) and non-dislocation (*n* = 104) groups. Demographic characteristics, injury mechanism, trauma energy, fracture morphology, posterior malleolar fracture classification, syndesmotic injury, and deltoid ligament injury were evaluated. Independent risk factors were identified using multivariate logistic regression analysis. Bone union time and postoperative osteoarthritis were also compared. **Results:** Haraguchi type II posterior malleolar fracture (*p* < 0.001) and syndesmotic injury (*p* = 0.017) were independently associated with ankle fracture–dislocation. Bone union took significantly longer in the dislocation group than in the non-dislocation group (7.04 ± 3.75 vs. 5.29 ± 3.48 months; *p* = 0.026). Postoperative osteoarthritis occurred more frequently in the dislocation group (42.3% vs. 24.0%), although the difference was not statistically significant (*p* = 0.063). **Conclusions:** Haraguchi type II posterior malleolar fracture morphology and syndesmotic injury were associated with ankle fracture–dislocation. These findings suggest that posteromedial structural disruption and syndesmotic instability play important roles in ankle fracture–dislocation. Furthermore, ankle fracture–dislocation was associated with delayed bone union, indicating a more severe injury pattern.

## 1. Introduction

Ankle fractures are among the most common injuries encountered in orthopedic practice [1]. Among ankle fractures, supination–external rotation (SER) injuries represent the most frequent ankle fracture pattern in the Lauge-Hansen (LH) classification system and are commonly associated with instability, as the posterior malleolus (PM) is affected [2,3].

Ankle fracture–dislocation is a relatively common injury pattern, occurring in approximately 21–50% of ankle fractures [4,5]. Previous studies have demonstrated that ankle fracture–dislocation is associated with higher rates of chronic pain, posttraumatic osteoarthritis, and worse functional outcomes compared with ankle fractures without dislocation [4,5,6]. Despite its clinical importance, the factors and mechanisms responsible for the development of dislocation remain incompletely understood [4,5]. Dislocation is believed to occur when multiple osseous and ligamentous stabilizers of the ankle are disrupted simultaneously [7]. To date, the stability of the ankle joint is known to depend on the integrity of three key components: the posterior column, the medial structures, and the syndesmotic complex [8].

Among them, the PM has received increased attention because of its important role in syndesmotic stability, rotational ankle stability, and maintenance of tibiotalar congruity [1]. Computed tomography (CT) is increasingly used to characterize PM fracture morphology and to facilitate preoperative planning, particularly in complex ankle fractures [1,9]. Several CT-based classification systems have been developed for PM fractures. Haraguchi and Bartoníček are among the most widely used morphology-based classification systems for PM fractures. The Bartoníček classification provides a detailed description of fracture morphology and has substantial potential for clinical decision making and research. In the present study, however, the Haraguchi classification was selected because one of its fracture patterns explicitly identifies posteromedial extension, which was central to our study hypothesis. In addition to the PM, the medial malleolus (MM), deltoid ligament complex, and syndesmotic ligaments contribute significantly to ankle stability [10]. Each of these components has been individually associated with ankle instability [10].

To our knowledge, few studies have investigated which specific patterns of structural injury contribute to the development of ankle fracture–dislocation. Previous studies have reported that ankle fracture–dislocation is more frequently associated with high-energy injury mechanisms and advanced rotational fracture patterns, particularly supination–external rotation stage III or IV injuries [4,5,11]. Trimalleolar fractures and PM involvement, including a larger PM, have also been observed more frequently in ankle fracture–dislocation patterns [5]. Disruption of the syndesmotic complex and medial stabilizing structures, including the deep deltoid ligament or MM, may further compromise tibiotalar stability and contribute to talar displacement [5,11,12]. However, most previous studies have focused on the incidence, treatment, or prognosis of fracture–dislocation within specific ankle fracture patterns, whereas the independent contributions of individual osseous and ligamentous injuries remain incompletely understood [2,4,5]. In particular, the relative contribution of the PM, MM, deltoid ligament complex, and syndesmotic complex to ankle fracture–dislocation has not been fully elucidated [2,5]. Furthermore, few studies have comprehensively evaluated the risk factors and radiological outcomes associated with ankle fracture–dislocation [6].

Therefore, the primary aim of this study was to identify demographic, injury-related, and structural factors associated with ankle fracture–dislocation. In particular, we investigated the influence of PM fracture morphology, medial and lateral malleolar fractures, syndesmotic injury, and deltoid ligament injury on the dislocation. The secondary aim was to compare radiographic outcomes, including time to bone union and radiographic osteoarthritic changes, between patients with and without dislocation.

We hypothesized that specific fracture patterns would be strongly associated with ankle fracture–dislocation and that patients with ankle fracture–dislocation would demonstrate worse radiographic outcomes than those without dislocation.

## 2. Materials and Methods

### 2.1. Study Design and Patient Selection

**Figure 1:**
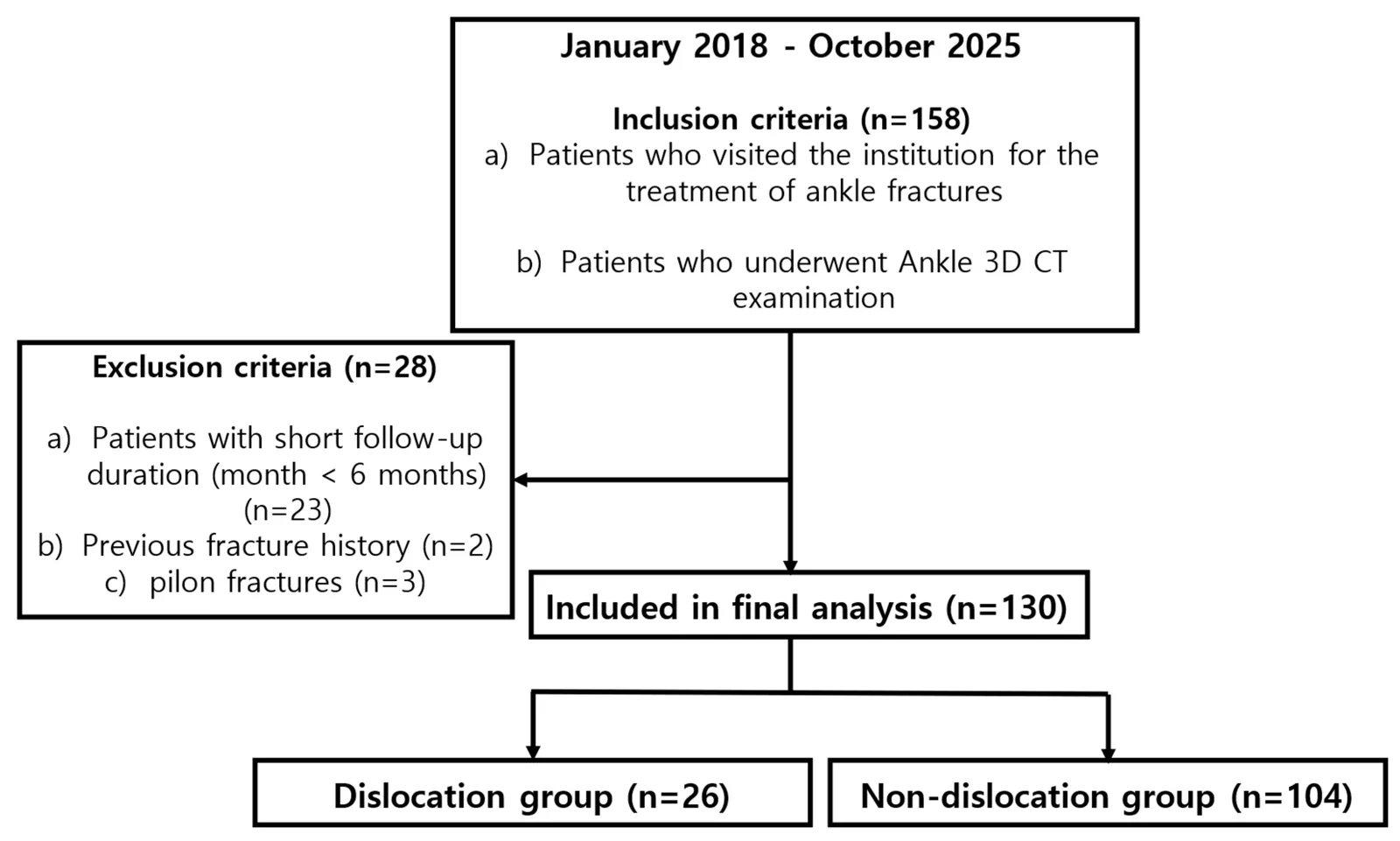
Flowchart of patient selection and study enrollment.

**Figure 2:**
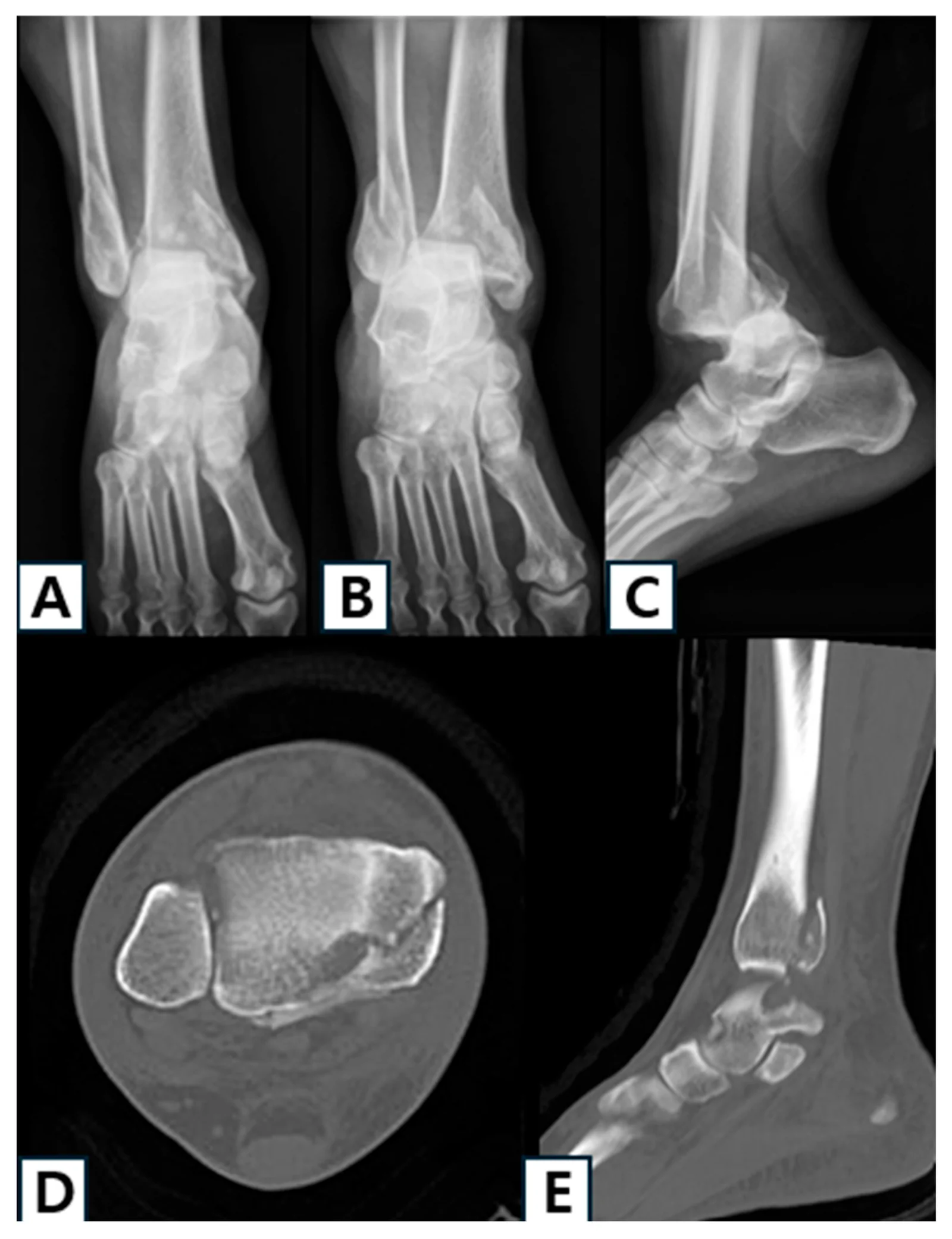
Radiographs and CT images of the right ankle in the dislocation group. (**A**) Anteroposterior, (**B**) mortise, and (**C**) lateral radiographs; (**D**) axial and (**E**) sagittal CT images.

**Figure 3:**
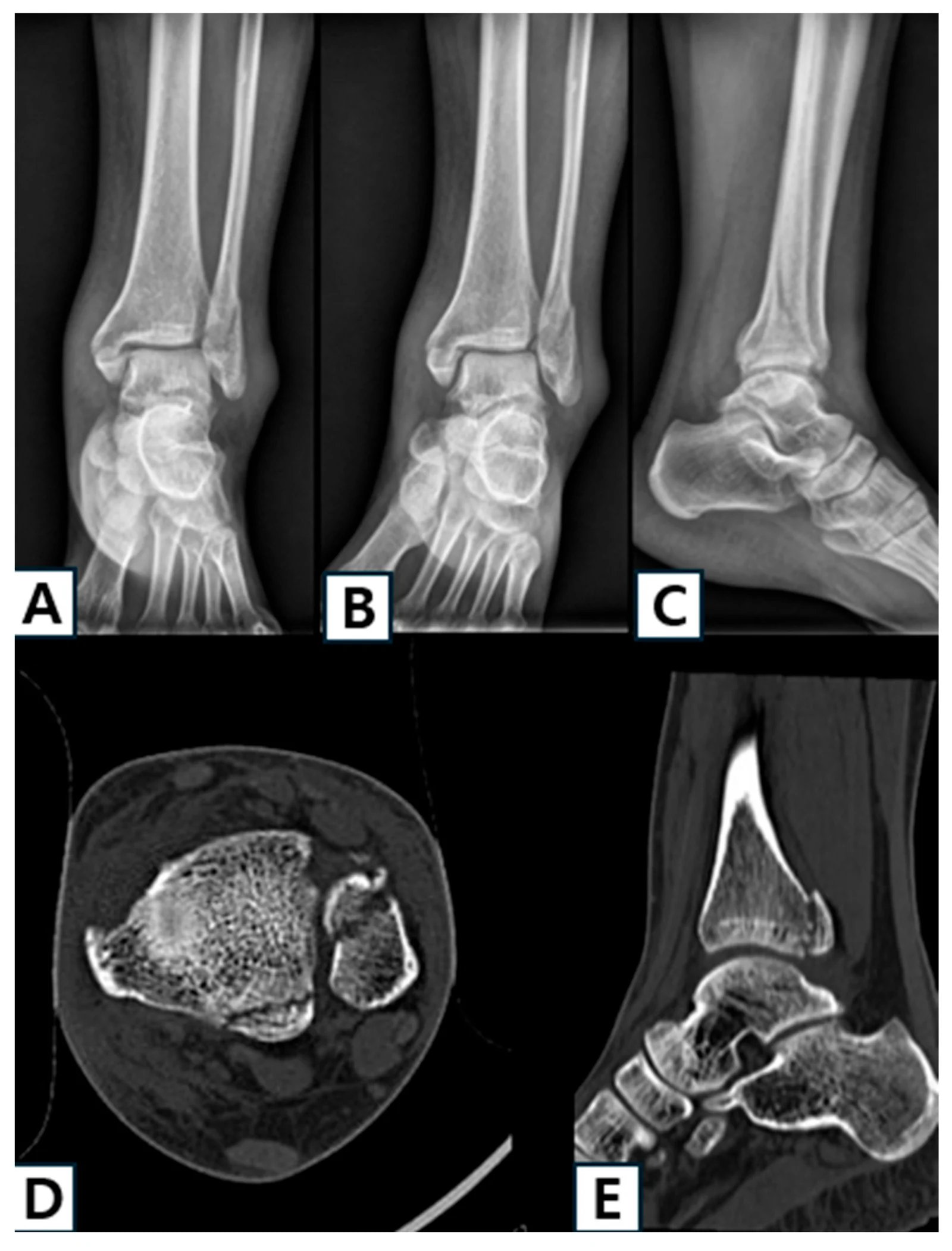
Radiographs and CT images of the left ankle in the non-dislocation group. (**A**) Anteroposterior, (**B**) mortise, and (**C**) lateral radiographs; (**D**) axial and (**E**) sagittal CT images.

This single-center retrospective cohort study was approved by the Institutional Review Board of Konyang University Hospital. From January 2018 to October 2025, patients who visited Konyang University Hospital for the operative treatment of ankle fractures and underwent perioperative simple ankle radiographs and preoperative ankle CT were included in this study (*n* = 158). The exclusion criteria included a follow-up duration of less than 6 months (*n* = 23), previous fracture history (*n* = 2), and pilon fractures (*n* = 3). Following the application of the exclusion criteria, 130 patients were included in the final analysis (Figure 1). Patients were categorized into two groups based on whether an ankle fracture–dislocation was present at the time of injury: the dislocation group (*n* = 26; Figure 2) and the non-dislocation group (*n* = 104; Figure 3).

The demographic and clinical data, including sex, age, obesity, smoking status, diabetes mellitus, injury mechanism, trauma energy at the time of injury, follow-up period, and diagnosis, were evaluated by reviewing the patients’ electronic medical records. Obesity was defined as a BMI ≥ 25 kg/m^2^ according to the Asia-Pacific criteria [13]. Injury mechanisms were categorized into slip-down injuries, falls from height, and direct trauma, including traffic accidents and sports injuries [5]. Trauma energy was categorized into low-energy and high-energy injuries. Low-energy injuries were defined as falls from standing height, whereas high-energy injuries included motor vehicle accidents or falls from height [14].

### 2.2. Surgical Technique

Surgical treatment was performed according to AO principles [15]. Fracture fixation was performed sequentially, beginning with fixation of the lateral and PM in the lateral decubitus position, followed by fixation of the MM after repositioning the patient supine. PM fixation was generally indicated when the fracture fragment involved ≥ 30% of the tibial plafond. For smaller fragments, fixation was not performed when satisfactory reduction was achieved through ligamentotaxis after lateral malleolar fixation and ankle joint stability was confirmed intraoperatively.

When PM fixation was required, the fragment was reduced using fracture reduction forceps. Whenever feasible, a small incision was made between the fibula and Achilles tendon, and percutaneous fixation was performed using one or two 4.0 mm cannulated screws. When adequate reduction and fixation could not be achieved using the percutaneous technique, an extended posterolateral approach was used between the fibula and Achilles tendon. The fracture site was then directly visualized, followed by open reduction and internal fixation.

After satisfactory reduction and fixation of the lateral and PM, the patient was repositioned supine. The MM fracture was reduced and fixed using one or two 4.0 mm cannulated screws. When a deltoid ligament injury was present, the ligament was repaired using absorbable sutures. Finally, syndesmotic stability was assessed using the bone hook test. When syndesmotic instability was identified, trans-syndesmotic fixation was performed using a 3.5 mm cortical screw with quadricortical purchase, placed 2–3 cm proximal to the ankle joint. The syndesmotic screw was removed 3–4 months after surgery.

### 2.3. Radiographic Evaluation

Plain radiographs and three-dimensional (3D) CT scans (TSX-303A, TOSHIBA, Otawara, Japan) were reviewed in all patients. Plain radiographs of the ankle, including anteroposterior (AP), lateral, and mortise views, were obtained preoperatively, postoperatively, and at each follow-up visit. Dislocation of the ankle was assessed on the initial radiographs obtained before reduction and was defined as no apposition of the tibia and talus on either the anteroposterior or lateral view [5]. The presence of MM fracture, syndesmotic injury, lateral malleolar fracture, and deltoid ligament injury were evaluated with X-ray and CT, and the results were classified with LH classification.

**Figure 4:**
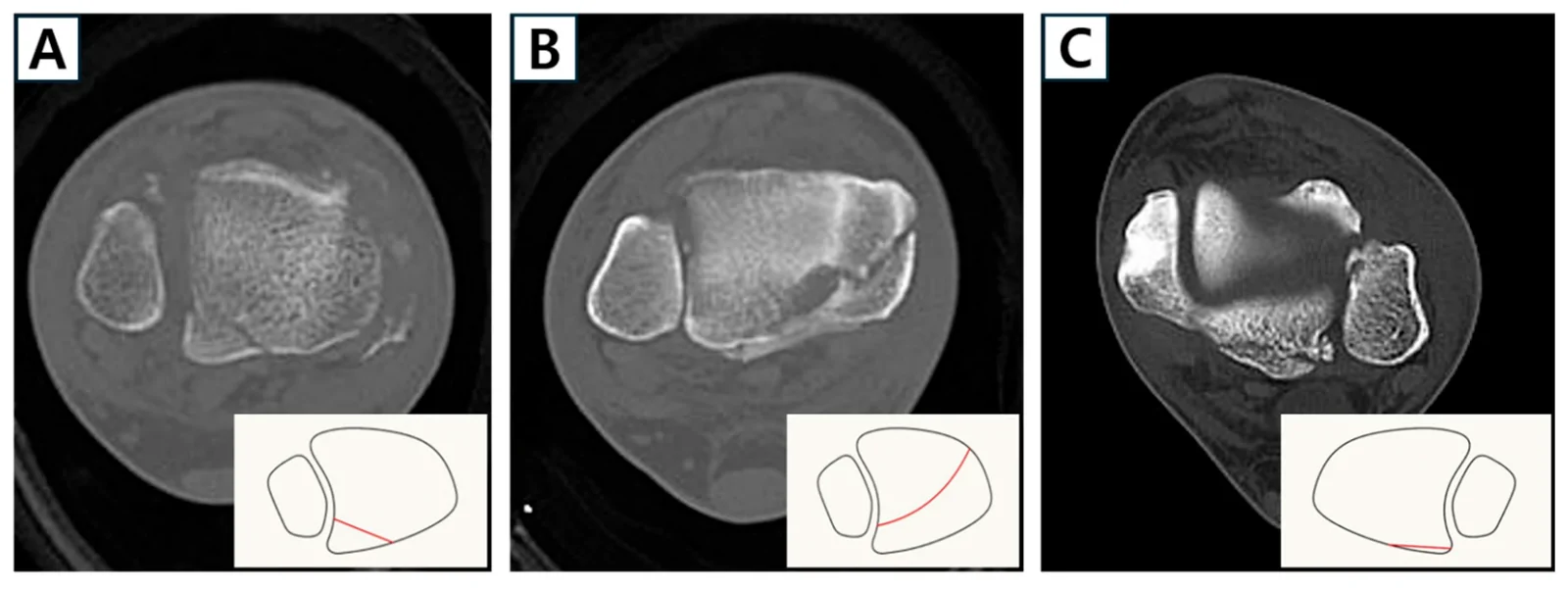
CT-based classification of posterior malleolar fractures according to the Haraguchi classification. (**A**) Haraguchi type I, wedge-shaped posterolateral fragment; (**B**) Haraguchi type II, medial-extension fracture; and (**C**) Haraguchi type III, small shell fracture.

PM fractures were classified according to the Haraguchi classification using ankle CT images. The Haraguchi classification categorizes PM fractures into three morphologic subtypes based on axial CT scans [9]. Type I fractures are wedge-shaped posterolateral fragments extending toward the fibular notch [6]. Type II fractures demonstrate a medial extension fracture line that creates a larger fragment involving both the posterolateral and posteromedial aspects of the distal tibia [6]. Type III fractures are small shell-type fractures involving only the posterior rim of the tibial plafond [6] (Figure 4).

In addition to the PM, the lateral malleolar, MM fracture, deltoid ligament injury, and syndesmotic injury were evaluated. Deltoid ligament injury was defined as widening of the medial clear space (>4 mm) on an ankle mortise radiograph [16]. Syndesmotic injury was defined as widening of the distal tibiofibular syndesmosis on an ankle mortise radiograph (tibiofibular clear space > 6 mm) [17]. Deltoid ligament and syndesmotic injuries were identified on the initial radiographs obtained before reduction rather than by intraoperative stress testing or stability assessment after fracture fixation. However, restoration of deltoid and syndesmotic stability was confirmed after deltoid ligament repair and syndesmotic positional screw fixation, respectively. The time to bone union and the presence of ankle osteoarthritis at the final follow-up were evaluated. Radiographic bone union was defined as obliteration of the fracture line with restoration of cortical continuity on follow-up anteroposterior, mortise, and lateral radiographs. In patients with bimalleolar or trimalleolar fractures, time to union was determined by the last fracture component to satisfy these radiographic criteria [9]. The development of radiographic osteoarthritic changes was defined as the presence of newly developed osteophytes in the tibiotalar joint on follow-up radiographs [18]. Two orthopedic surgeons with more than 10 and 5 years of clinical experience, respectively, independently reviewed all radiographs and CT images to classify ankle fracture morphology. To assess interobserver reliability, all radiographic parameters for the 130 cases were measured independently and without conferring by the two authors. The interobserver reliabilities were expressed in terms of the intraclass correlation coefficient (ICC). All radiographic measurements showed excellent (ICC > 0.8) interobserver reliabilities in the present study.

### 2.4. Statistical Analysis

An a priori power analysis was performed using the G*Power software (version 3.1.9.7; Heinrich Heine University, Düsseldorf, Germany) based on the difference in the proportion of Haraguchi type II posterior malleolar fractures between the dislocation and non-dislocation groups (61.5% vs. 12.5%). Assuming a two-sided α level of 0.05, a statistical power of 90%, and an allocation ratio of 4:1, the minimum required sample size was calculated to be 60 patients (12 in the dislocation group and 48 in the non-dislocation group). The final study cohort comprised 130 patients (26 and 104 patients, respectively), exceeding the required sample size.

Statistical analyses were performed with SPSS version 22 for Windows (IBM Corp., Armonk, NY, USA). Categorical variables were compared using the chi-square test or Fisher’s exact test, as appropriate. Continuous variables were compared using the independent-samples *t*-test or Mann–Whitney U test, according to data distribution assessed using the Shapiro–Wilk test.

To identify independent risk factors associated with ankle fracture–dislocation, variables associated with ankle fracture–dislocation in univariate analyses at *p* < 0.05 were considered for multivariate binary logistic regression. Conceptually overlapping variables were not entered simultaneously to reduce redundancy and potential multicollinearity. Accordingly, Haraguchi type II fracture, MM fracture, and syndesmotic injury were included in the final model. Odds ratios (ORs) and 95% confidence intervals (CIs) were calculated. Statistical significance was defined as *p* < 0.05.

## 3. Results

### 3.1. Demographic Data

There were no significant differences between the two groups with respect to sex, age, obesity, smoking status, diabetes mellitus, injury mechanism, trauma energy at the time of injury, and follow-up periods (Table 1). The mean follow-up periods were 17.59 ± 10.86 months in the dislocation group and 19.19 ± 9.79 months in the non-dislocation group, with no significant difference between the groups (*p* = 0.493).

### 3.2. Radiographic Data

Among the 26 patients with ankle fracture–dislocation, 25 (96.2%) had posterior dislocation of the talus, and one (3.8%) had anterior dislocation. Because only one anterior dislocation was identified, a comparative analysis according to dislocation direction was not performed.

PM fractures were present in 96.2% of patients in the dislocation group and 53.8% of those in the non-dislocation group (*p* < 0.001) (Table 2). MM fractures were also more frequent in the dislocation group (92.3% vs. 63.5%; *p* = 0.004). Consequently, medial injury, defined as either an MM fracture or deltoid ligament injury, was identified in 100% and 72.1% of patients, respectively (*p* = 0.004). Syndesmotic injury occurred in 53.8% of the dislocation group compared with 26.9% of the non-dislocation group (*p* = 0.009).

There was no significant difference in the prevalence of Haraguchi type I fractures between the two groups (30.8% vs. 30.8%; *p* = 1.000). In contrast, Haraguchi type II fracture demonstrated a strong association with dislocation. The incidence of dislocation was significantly higher in patients with type II fractures (61.5% vs. 12.5%; *p* < 0.001). There was no significant difference in the prevalence of Haraguchi type III fractures between the two groups (3.8% vs. 10.6%; *p* = 1.000).

In the LH classification, no SER type II injuries were observed in the dislocation group, whereas 16 cases were identified in the non-dislocation group (*p* = 0.023). In contrast, SER type IV injuries were more frequent in the dislocation group than in the non-dislocation group (26.6% vs. 13.6%) but were not statistically significantly associated (*p* = 0.065).

The mean time to bone union was significantly longer in the dislocation group than in the non-dislocation group (7.04 ± 3.75 vs. 5.29 ± 3.48 months; *p* = 0.026). Postoperative osteoarthritis occurred more frequently in the dislocation group (42.3% vs. 24.0%); however, this difference did not reach statistical significance (*p* = 0.063). Open fracture was rare in both groups, each having two patients for dislocation and one patient for non-dislocation, and no statistically significant association was observed between open fracture and dislocation.

Variables showing a significant association with ankle fracture–dislocation in the univariate analyses (Haraguchi type II fracture, MM fracture, and syndesmotic injury) were entered into the multivariate binary logistic regression model (Table 3). Haraguchi type II fracture (OR: 9.22; 95% CI: 3.05–27.90; *p* < 0.001) and syndesmotic injury (OR: 3.55; 95% CI: 1.25–10.05; *p* = 0.017) were independently associated with ankle fracture–dislocation. MM fracture was not independently associated with dislocation after adjustment (OR: 2.59; 95% CI: 0.51–13.16; *p* = 0.253).

## 4. Discussion

The main finding of the present study is that Haraguchi type II fracture and syndesmotic injury were associated with ankle fracture–dislocation. In addition, the dislocation group showed a significantly longer time to bone union than those without dislocation. These findings suggest that anatomical fracture patterns play a key role in the development and prognosis of ankle fracture–dislocation.

Compared with ankle fractures without dislocation, fracture–dislocation injuries are more unstable and have been associated with prolonged recovery, posttraumatic osteoarthritis, and poor functional outcomes [5,19]. Although ankle fracture–dislocation has been regarded as a consequence of high-energy trauma or severe rotational force, the anatomical factors contributing to dislocation have received relatively less attention [5,11,20,21]. Previous CT-based studies have demonstrated that PM fracture morphology is associated with the injury mechanism, patterns of concomitant medial and syndesmotic injuries, and clinical outcomes [12,20,22]. However, whether posteromedial extension, medial-sided injury, and syndesmotic disruption are independently associated with ankle fracture–dislocation remains unclear [5]. Therefore, this study focused on identifying structural factors associated with ankle fracture–dislocation and evaluating which ankle stabilizing structures are most closely related to instability.

Recent studies have suggested that PM fracture morphology may have greater clinical relevance than fragment size alone in determining ankle stability [23,24]. The findings of this study are consistent with this concept. Multivariate analysis demonstrated that Haraguchi type II morphology was independently associated with ankle fracture–dislocation. Haraguchi type II fractures are characterized by a posteromedial extension fracture line connecting the PM to the medial side of the distal tibia [6]. MM fracture was significantly associated with ankle fracture–dislocation in the univariate analysis but did not remain an independent predictor after adjustment in the multivariate model. Together, these findings suggest that a PM fracture extending to the MM, rather than being the simple coexistence of posterior and medial injuries, may be more important in the development of ankle fracture–dislocation. Previous studies have shown that the morphology of PM fractures, especially those extending into the posteromedial region, is linked to syndesmotic instability, joint incongruity, and poorer clinical outcomes [12,25,26]. A possible explanation for our findings lies in the medial stabilizing anatomy of the ankle. Posteromedial fracture patterns frequently include the posterior colliculus of the MM [6], which provides the insertion for the deep posterior tibiotalar ligament [18,27]. Because this ligament plays an important role in limiting posterior talar translation and external rotation, injury involving the posterior colliculus may weaken the deep medial stabilizing structures [27]. However, the present study did not directly evaluate injury to individual deltoid ligament components. Therefore, the contribution of the deep posterior tibiotalar ligament should be regarded as a possible anatomical explanation rather than a mechanism demonstrated by the present findings.

Also, the Haraguchi classification primarily describes the location and orientation of the posterior malleolar fracture line and does not fully account for other potentially relevant morphological characteristics. Fragment size, incisural involvement, articular impaction, posterior plafond involvement, and fragment displacement were not evaluated in the present study. Therefore, although Haraguchi type II morphology remained associated with ankle fracture–dislocation after adjustment for the measured variables, the present findings cannot determine whether this fracture pattern itself represents the relevant morphological feature or whether it serves as a surrogate for a larger, more displaced, or otherwise more severe PM injury. Accordingly, the observed association should be interpreted as reflecting a broader posteromedial injury pattern rather than establishing an independent causal role of Haraguchi type II morphology.

Another important finding of this study was that syndesmotic injury remained an independent factor for ankle fracture–dislocation in a multivariate logistic regression model. This result suggests that ankle stability depends not only on the integrity of osseous structures but also on the syndesmotic ligament complex. Interestingly, lateral malleolar fracture was not significantly associated with ankle fracture–dislocation, whereas syndesmotic injury remained significant. This finding indicates that syndesmotic injury represents functional disruption of the ankle mortise and may, therefore, contribute more directly to dislocation. Among the 26 patients with ankle fracture–dislocation, 25 (96.2%) had posterior dislocation of the talus, indicating that posteriorly directed displacement was the predominant pattern in this cohort. Nevertheless, because only one patient had anterior dislocation, and the posterior dislocations could not be consistently subclassified as posteromedial or posterolateral, the present data do not establish a direct relationship between Haraguchi type II morphology and a specific direction of talar displacement. Severe rotational or compressive forces may produce posterior talar displacement together with posteromedial PM fracture extension and syndesmotic injury; however, this proposed sequence remains speculative. Overall, the strong associations of Haraguchi type II morphology and syndesmotic injury with ankle fracture–dislocation suggest that both the posteromedial osseous structures and the syndesmotic complex may contribute importantly to ankle stability during high-energy injury.

In the Lauge-Hansen classification, no SER type II injuries were identified in the dislocation group, whereas SER type IV injuries accounted for most dislocation cases (65.4%). This distribution is consistent with the sequential nature of the SER injury mechanism. SER type II injuries are generally limited to the syndesmotic and lateral structures, whereas progression to SER type III or IV involves posterior and medial stabilizing structures. Previous studies have reported poorer outcomes and a greater incidence of instability or dislocation in more advanced rotational fracture patterns. Therefore, our findings should not be interpreted as demonstrating that SER type II morphology is independently protective; rather, they support the broader concept that combined disruption of posterior, medial, and syndesmotic structures is associated with ankle fracture–dislocation.

The dislocation group demonstrated a significantly longer time to bone union than the non-dislocation group, suggesting that ankle fracture–dislocation represents a more severe injury pattern. Although the prevalence of osteophyte formation did not differ significantly between the two groups, its incidence was nearly twofold higher in the dislocation group. This finding is consistent with previous studies reporting that ankle fracture–dislocations are associated with an increased risk of posttraumatic osteoarthritis and worse radiologic outcomes [5].

Interestingly, age, DM, injury mechanism and trauma energy level were not significantly associated with dislocation in this study. Although ankle fracture–dislocation has traditionally been regarded as a high-energy injury pattern [4,5], the absence of a significant statistical association between trauma energy level and dislocation suggests that structural injury characteristics may be more relevant determinants of dislocation than injury mechanism alone. These indicate that the pattern and extent of stabilizing structure disruption may play a greater role in the development of ankle fracture–dislocation than the type and magnitude of external force itself.

Overall, the clinical implications of our findings are as follows. Because Haraguchi type II PM fractures and syndesmotic injuries were identified as important factors associated with ankle fracture–dislocation, preoperative CT evaluation should be considered in patients presenting with ankle dislocation, regardless of whether successful reduction has already been achieved. Particular attention should be paid to posteromedial extension of the PM fracture on axial CT images and to radiographic features suggestive of syndesmotic injury, as these findings may influence preoperative planning and the surgical strategy. An analysis of the operative procedures performed in the 26 patients with ankle fracture–dislocation showed plate fixation of the lateral malleolus in 24 patients, screw fixation of the PM in nine, screw fixation or tension-bend wiring of the MM in 24, and syndesmotic screw fixation in seven. Despite the high prevalence of Haraguchi type II fractures in the dislocation group, direct fixation of the PM fragment was required less frequently than expected. This finding suggests that secure fixation of the lateral and MM may restore stability to the posteromedial structures sufficiently in selected patients, provided that satisfactory ankle stability is confirmed intraoperatively. Therefore, the presence of a Haraguchi type II fracture in an ankle fracture–dislocation may not necessarily mandate an additional or novel surgical approach specifically targeting the posteromedial fragment. Rather, adherence to established fixation principles, including accurate reduction and stable fixation of the lateral and MM, remains essential for restoring the stability of the posteromedial ankle complex. None of the 26 patients with ankle fracture–dislocation experienced complications related to residual ankle instability through the final follow-up after treatment using our surgical strategy. Notably, none of the 26 patients underwent temporary external fixation, and all definitive surgical procedures were performed within 1–3 days after injury.

To our knowledge, this is one of the first studies to comprehensively evaluate independent risk factors for ankle fracture–dislocation using multivariate logistic regression analysis. Unlike previous studies that primarily focused on clinical outcomes after fracture–dislocation [4,5,12], the present study investigated the structural factors contributing to the occurrence of dislocation itself. In particular, PM fractures were analyzed according to the CT for Haraguchi classification, and MM fracture, lateral malleolar fracture, syndesmotic injury, and deltoid ligament injury were comprehensively evaluated to determine their respective contributions to ankle fracture–dislocation.

However, the limitation of this study was that it was a retrospective study conducted at a single institution with a relatively small sample size. The number of ankle fracture–dislocations was relatively small, with only 26 outcome events available for the multivariable logistic regression analysis. Although the final model was restricted to three variables and no potentially relevant multicollinearity was identified, the number of events per variable was limited. The adjusted associations should consequently be regarded as exploratory rather than definitive and require external validation in a larger cohort. Second, clinical outcomes were not assessed using validated functional outcome measures, such as the Patient-Reported Outcomes Measurement Information System score. Third, only patients who underwent preoperative CT were included. Because CT is more frequently obtained for complex or unstable fracture patterns, this inclusion criterion may have introduced selection bias toward more severe injuries and may limit the generalizability of our findings to all ankle fractures. Fourth, the morphological assessment focused primarily on fracture classification and did not include fragment size, incisural involvement, articular impaction, posterior plafond involvement, or fragment displacement. These unmeasured characteristics may have differed between the groups and may partly explain the observed association between Haraguchi type II morphology and ankle fracture–dislocation. Therefore, residual confounding by fracture severity cannot be excluded, and the present study cannot establish whether Haraguchi type II morphology itself or its accompanying morphological characteristics are more directly related to fracture–dislocation. In addition, although 25 of the 26 fracture–dislocations were posteriorly directed, further subclassification into posteromedial and posterolateral dislocation patterns was not consistently possible because of the retrospective study design and the available radiographic images. Therefore, fracture morphology could not be compared according to specific dislocation direction, and the proposed pathomechanical relationship between posteromedial structural disruption and fracture–dislocation remains speculative. Lastly, the present study did not evaluate whether these findings should influence surgical indications, fixation strategies, or postoperative treatment protocols. Future studies with larger cohorts and longer follow-up are warranted to validate these findings and individualize the treatment strategies based on fracture morphology.

## 5. Conclusions

Haraguchi type II PM fractures and syndesmotic injury were associated with ankle fracture–dislocation. These findings suggest that posteromedial structural disruption, particularly a Haraguchi type II fracture accompanied by syndesmotic injury, may be an important feature of ankle fracture–dislocation. Ankle fracture–dislocation was also associated with delayed bone union, reflecting a more severe injury pattern. Furthermore, neither injury mechanism nor trauma energy level was significantly associated with ankle fracture–dislocation, suggesting that intrinsic anatomic and structural instability may play a greater role in its development than the external injury pattern or magnitude of force.

Prospective studies with standardized follow-up intervals, as well as cross-sectional long-term follow-up of the current cohort, are needed to validate these findings, reduce follow-up-related bias, and evaluate clinical and radiographic outcomes more reliably.

## Figures and Tables

**Table 1:** Demographic data of the present study.

	Dislocation (*n* = 26)	Non-Dislocation (*n* = 104)	*p*-Value
Male (Sex)	12 (46.2%)	44 (42.3%)	0.723
Age (years) ^a^	50.04 ± 18.31	49.83 ± 18.05	0.958
Obesity ^b^	5 (19.2%)	17 (16.3%)	0.661
Smoking	9 (34.6%)	20 (19.2%)	0.092
Diabetes mellitus	5 (19.2%)	23 (22.1%)	0.749
Injury mechanism			
Slip-down injury	15 (57.7%)	67 (64.4%)	0.650
Fall from height	3 (11.5%)	6 (5.8%)	0.382
Direct trauma	8 (30.8%)	31 (29.8%)	0.123
High-energy trauma	10(38.5%)	36 (34.6%)	0.714
Follow-up period (months) ^a^	17.59 ± 10.86	19.19 ± 9.79	0.493

^a^ Values are presented as means ± standard deviation. ^b^ Defined as a body mass index of ≥25 kg/m^2^. Values are presented as frequencies (%).

**Table 2:** Radiographic data of the present study.

		Dislocation (*n* = 26)	Non-Dislocation (*n* = 104)	*p*-Value
	Posterior malleolar fracture	25 (96.2%)	56 (53.8%)	<0.001 ***
	Lateral malleolar fracture	26 (100%)	93 (89.4%)	<0.120
	Medial malleolar fracture	24 (92.3%)	66 (63.5%)	0.004 **
	Deltoid ligament injury	4 (15.4%)	13 (12.5%)	0.748
	Medial malleolar fracture or deltoid ligament injury	26(100%)	75 (72.1%)	0.004 **
	Syndesmotic injury	14(53.8%)	28(26.9%)	0.009 **
PM fracture classification				
	PM fracture (-)	1 (3.8%)	48 (46.2%)	<0.001 ***
	Haraguchi type I	8 (30.8%)	32 (30.8%)	1.000
	Haraguchi type II	16 (61.5%)	13 (12.5%)	<0.001 ***
	Haraguchi type III	1 (3.8%)	11 (10.6%)	1.000
Lauge-Hansen type				
	SER type I	0 (0%)	2 (1.9%)	1.000
	SER type II	0 (0%)	16 (15.4%)	0.023 *
	SER type III	0 (0%)	2 (1.9%)	1.000
	SER type IV	17 (65.4%)	47 (45.2%)	0.065
	SA type I	0 (0%)	7 (6.7%)	0.344
	SA type II	2 (7.7%)	10 (9.6%)	0.693
	PA type I	0 (0%)	1(1.0%)	1.000
	PER type I	0 (0%)	1(1.0%)	1.000
	PER type II	0 (0%)	1(1.0%)	1.000
	PER type IV	7 (26.9%)	16 (15.4%)	0.248
Time to bone union (months) ^a^		7.04 ± 3.75	5.29 ± 3.48	0.026 *
Osteoarthritis		11 (42.3%)	25 (24.0%)	0.063

Values are presented as frequencies (%). ^a^ Values are presented as means ± standard deviation. PM, posterior malleolar; SER, supination–external rotation; SA, supination–adduction; PA, pronation–abduction; PER, pronation–external rotation. * *p* < 0.05, ** *p* < 0.01, and *** *p* < 0.001.

**Table 3:** Injury characteristics associated with ankle fracture–dislocation.

	Odds Ratio	CI 95%	*p*-Value
Haraguchi type II	9.22	3.05–27.90	<0.001 ***
Syndesmotic injury	3.55	1.25–10.05	0.017 *
Medial malleolus	2.59	0.51–13.16	0.253

* *p* < 0.05; *** *p* < 0.001.

## Data Availability

The data presented in this study are available in this article.

## Abbreviations

The following abbreviations are used in this manuscript:

SER	Supination–external rotation
LH	Lauge-Hansen
PM	Posterior malleolus
CT	Computed tomography
MM	Medial malleolus
AP	Anteroposterior
ORs	Odds ratios
CIs	Confidence intervals
